#  Intra-accumbal Orexin-1 Receptors are Involved in Antinociception Induced by Stimulation of the Lateral Hypothalamus in the Formalin Test as an Animal Model of Persistent Inflammatory Pain

**Published:** 2016

**Authors:** Mahboubeh Jahangirvand, Fatemeh Yazdi, Marzieh Moradi, Abbas Haghparast

**Affiliations:** Neuroscience Research Center, School of Medicine, Shahid Beheshti University of Medical Sciences, PO Box 19615-1178, Tehran, Iran.

**Keywords:** Pain, Orexin-1 receptor; SB334867, Lateral hypothalamus, Nucleus accumbens, Formalin test, Rat

## Abstract

Orexin, mainly produced by orexin-expressing neurons in the lateral hypothalamus (LH), plays an important role in pain modulation. Moreover, it is shown that the nucleus accumbens (NAc) is one of the important areas involved in this modulation. Orexin-1 (OX1) and orexin-2 (OX2) receptors are densely distributed in the NAc. The study investigated the involvement of OX1 receptors in the NAc on antinociception induced by intra-LH administration of carbachol in formalin test. Rats were unilaterally implanted by two separate cannulae into the LH and NAc. Different doses of SB334867, as an OX1 receptor antagonist, were microinjected into the NAc (1, 3 and 10 nM/0.5 µL DMSO) prior to intra-LH carbachol injection (250 nM/0.5 µL saline). Formalin test was applied as an animal model of persistent inflammatory pain. The animals received a subcutaneous injection of formalin into the hind paw, 5 min after SB334867 administration. Pain scores were calculated at 5-min blocks for a 60-min test period. Results showed that the administration of SB334867 into the NAc decreased LH chemical stimulation-induced antinociception dose-dependently in early and second phase of formalin test. Our findings showed that OX1 receptors in the NAc may be involved in modulation of inflammatory pain.

## Introduction

Pain is a multidimensional experience that involves the interventions of brain areas that subserve sensory, affective and cognitive components (1). Pain is defined as “an unpleasant sensory and emotional experience associated with actual or potential tissue damage,” or described in terms of such damage (2). For defensing of such damage, there is a descending pain modulatory circuitry, including inhibitory and excitatory, which is used in this situation (3). In this modulatory system many areas of the brain and neurotransmitters would take a part. One neurotransmitter which is shown to be involved in pain modulation is the orexin (4-6).

Orexin was described as two distinct peptides – orexin A and orexin B; which also called hypocretin-1 and -2, composed by 33 and 28 amino acids, respectively. Orexin acts through two G-protein coupled receptors named orexin-1 receptor (OX1) and orexin-2 receptor (OX2) (7, 8). The Orexin A has a tendency to both OX1 and OX2 receptors, however the orexin B is selective for the OX2 receptors (8). Lateral hypothalamus (LH) is a major locus of the orexin-expressing neurons (7). The orexinergic system takes part in many physiological processes, such as feeding, arousal, sleep, reward, addiction, stress, and pain (9-12) and they do their physiological role through their receptors which are distributed in many brain areas like thalamus, basal ganglia, brain stem nuclei and spinal cord (7, 13-15). Several studies demonstrated that carbachol (a cholinergic agonist) infusion into the LH induces antinociception in both acute and tonic models of pain in rats. These studies evidenced that stimulation of orexinergic neurons on the LH induces an antinociceptive effect via several brain regions, such as the nucleus accumbens (NAc) and the ventral tegmental area (VTA) (4, 5, 11, 16). The NAc is a collection of neurons that forms the main part of the ventral striatum, which takes part on pain modulation (17, 18). Additionally, both OX1 and OX2 receptors have been reported to be expressed in the NAc (19). Intra-VTA and -accumbal injections of SB334867, an OX1r antagonist, or TCS OX2 29, an OX2r antagonist, prevented the antinociception induced by LH carbachol stimulation in tail-flick test (4, 5). In order to enlighten the post-synaptic role of LH orexin system on NAc OX1 receptors, the current study used formalin test as a model of inflammatory pain in rats to investigate the involvement of the OX1 receptors within the NAc on the antinociception induced by intra-LH injection of carbachol.

## Materials and Methods

*Animals*

Forty eight adult male albino Wistar rats (Pasteur Institute, Tehran, Iran) weighing 200–250 g were used in these experiments. The rats were acclimated to the vivarium (a 12 h light/dark cycle at a temperature controlled room, 23 ± 1 °C), for at least one week prior to the onset of the experiments, with free access to chow and tap water. The animals were randomly allocated to different experimental groups. Each animal was used only once. All experiments were executed in accordance with the Guide for the Care and Use of Laboratory Animals (National Institutes of Health Publication No.80-23, revised 1996) and were approved by the Research and Ethics Committee of Shahid Beheshti University of Medical Science.

*Stereotaxic surgery*

Rats were anesthetized with intraperitoneal (i.p.) injection of ketamine (100 mg/Kg) and xylazine (10 mg/Kg). Cannulae (23-gauge diameter, 11 mm long) were stereotaxically (stereotaxic apparatus, Stoelting, USA) implanted into the LH and NAc. The coordinates for these regions were determined by the rat brain atlas of Paxinos and Watson (2007) as AP = 2.6 ±0.15 mm caudal to the bregma, Lat = ±1.4 mm lateral to midline and DV = 8.5 mm ventral from the skull surface for the LH and for the NAc was AP = 1.6 ± 0.15 mm rostral to the bregma, Lat = ±1.6mm lateral to midline, DV = 7.8 mm ventral from the skull surface (20). The guide cannulae were secured in place using two stainless steel screws anchored to the skull and dental acryl cement. After the cement was completely dried and hardened, two stainless steel stylets inserted into the each guide cannulae to prevent occlusion during the recovery period. Then, animals were housed randomly and allowed to recover for a period of 5–7 days before the experiments.

*Drugs*

All drugs were freshly prepared on the day of the experiment. Carbachol (Carbamoylcholine chloride; Tocris Bioscience, Bristol, UK) as a cholinergic agonist dissolved in physiological saline and SB334867 (Tocris Bioscience, Bristol, UK); as an OX1 receptor antagonist dissolved in 12% dimethyl sulfoxide (DMSO; Sigma–Aldrich, Germany). 2.5%formalin was prepared by diluting 37% formaldehyde (i.e., the commercially available saturated aqueous solution of formaldehyde, Merk, Germany) with physiological saline solution.

*Drug administration*

Microinjections were performed by lowering a stainless steel injector cannula (30-gauge needle) with a length of 1 mm longer than the guide cannulae into the LH and NAc. The injector cannula was attached to a 1-µL Hamilton syringe by polyethylene tubing (PE-20), subsequently the drug solution or vehicles was infused unilaterally over 60s and was left for an additional 60s to prevent the backflow of drugs. Carbachol or saline were administered in a total volume 0.5 µL into the LH and SB334867 or 12% DMSO were administered in a total volume of 0.5 µL into the NAc. 

*Formalin test*

All rats were injected subcutaneously into the plantar surface of the right hind paw by formalin (2.5%; 50 µL), 5 min after microinjection of either the drugs or vehicles into the LH or NAc. Then placed in a 35 × 35 × 35 cm sized transparent plastic chamber with a mirror angled at 45˚ behind the box which used for observing the rat’s behavior during the test. Nociceptive behaviorswere observed in these experiments for 60-min period following formalin injection. During this time, pain scores were determined for every 5-min block by measuring the amount of time spent in each type of the four behavioral categories: 0, the position and posture of the injected hind paw was indistinguishable from another hind paw; 1, the injected paw had little or no weight on it; 2, the injected paw was elevated and did not have contact with any surface; 3, the injected paw was licked, bitten or shaken (18, 21). Then, a weighted nociceptive score, with a range of 0-3, was calculated by multiplying the time spent in each category by the category weigh, summing these products and dividing by the total time (300 s) for each 5-min block of time: 

Pain score = (t0 × 0) + (t1 × 1) + (t2 × 2) + (t3 × 3)/t0+t1+t2+t3

Formalin injection provokes a biphasic nociceptive response. The first 5-min block is considered as early phase (0–5 min), and the late phase takes more time and is defined from 15 to 60min after formalin injection into the hind paw. By utilizing this method, an ordinal scale (18) of nociceptive scores was generated with a range of 0–3.

*Experimental design*

The dose–response effects of intra-LH administration of carbachol has been published in our laboratory`s previous work (21). In that study carbachol microinjected to the LH in four doses (62.5, 125, 250 and 500 nM/0. 5 µL saline) 5 min before injection of formalin. The important result of the mentioned study was that intra-LH carbachol dose-dependently could block the nociceptive responses induced by formalin injection in both phases and the maximum effect was seen at the doses of the 250 and 500 nM. Accordingly, in this study, we used the dose of 250 nM.

Effect of intra-accumbal administration of OX1 receptor antagonist on antinociception induced by chemical stimulation of the LH

To examine the role of intra-accumbal OX1 receptors in the LH stimulation-induced antinociception, different doses of SB334867 (1, 3 and 10 nM/0.5µL DMSO) were unilaterally injected into the NAc, 5min prior to intra-LH administration of carbachol (effective dose; 250 nM/0.5 µL) and then test for formalin test. In the carbachol-control group rats received 12% DMSO instead of SB334867 and vehicles-control group, received 12% DMSO and saline (0.5 µL) into the NAc and LH, respectively.

*Histological verification*

**Figure 1:**
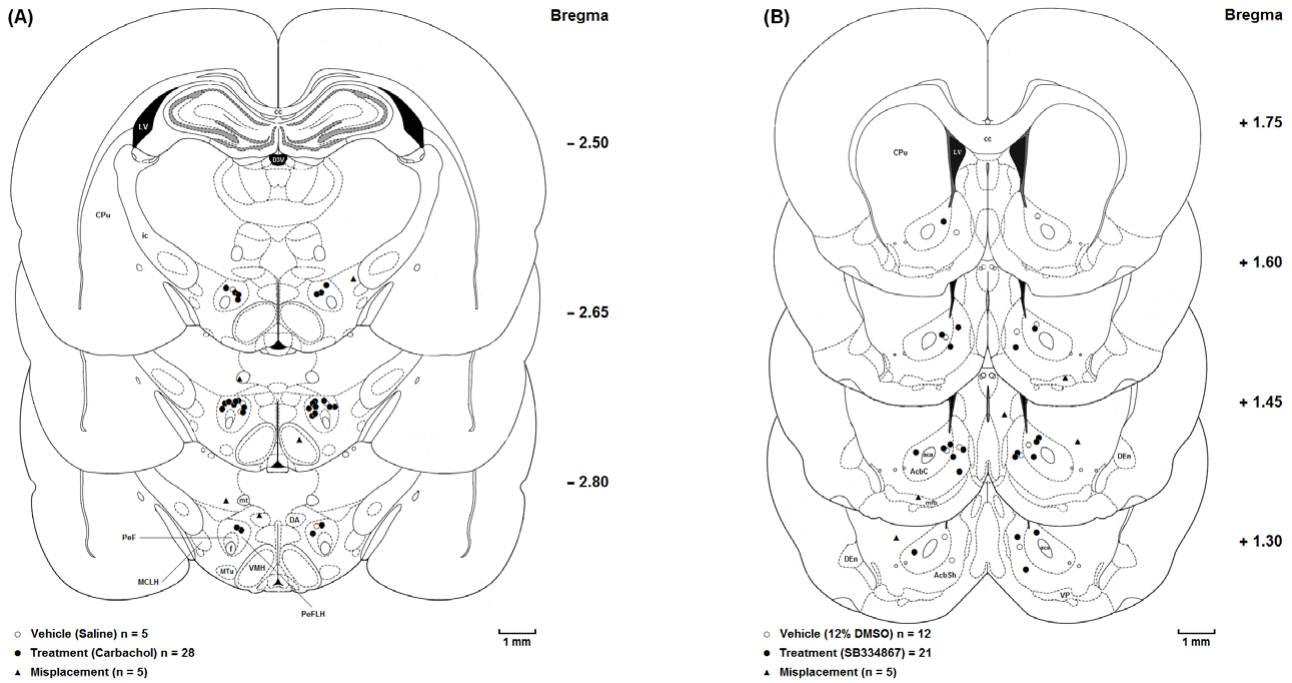
Coronal schematic sections show the microinjection sites in the *(A)* Lateral hypothalamus (○ Vehicle (Saline); ● Treatment (Carbachol); ▲ Misplacement) and*(B)* Nucleus accumbens (○Vehicle (12%DMSO); ● Treatment (SB334867); ▲ Misplacement). aca, anterior commissure, anterior part; AcbC, Accumbens nucleus, Core; AcbSh,Accumbens nucleus, Shell; cc, Corpus callosum; CPu, Caudate putamen (striatum); D3V, Dorsal 3rd ventricle; DA, Dorsal hypothalamic area; DEn, Dorsal endopiriform nucleus; f, fornix; ic, Internal capsule; LV, Lateral ventricle; MCLH, Magnocellular nucleus of the lateral hypothalamus; mfb, Medial forebrain bundle; mt, mammillothalamic tract; MTu, Medial tuberal nucleus; PeF, Perifornical nucleus; PeFLH, Perifornical part of lateral hypothalamus; VMH, Ventromedial hypothalamic nucleus; VP, Ventral pallidum

The animals were deeply anesthetized with ketamine and xylazine (100 mg/Kg and 10 mg/Kg, respectively) after the experiments were accomplished. Transcardial perfusion was carried out with 0.9% saline and 10% formalin solution. The brains were removed and cut coronally in 50-µM sections through the cannulae placements. The neuroanatomical locations of cannulae tips were confirmed using rat brain atlas (20). Only the results from animals with correct cannulae placements were included in the data analysis (Figure 1.).

*Statistics*

The obtained data are expressed as mean ± SEM (standard error of mean). To specify the treatment and time effects on the pain behaviors, the mean nociceptive scores of different groups were compared by two-way ANOVA followed by Bonferroni’s multiple comparisons test. In order to evaluate the nociceptive responses, area under the curve (AUC) was calculated as raw pain scores × time by linear trapezoidal method and a single value was used in statistical analyses. We calculated the AUC of the early phase (1-5 min) and late phase (15-60 min) separately. The calculated AUC values in all experimental groups were subjected to one-way ANOVA followed by protected Newman–Keuls test for multiple comparisons. One-way ANOVA followed by Dunnett›s test was used to calculate the percentage decrease of the AUC values calculated for pain scores in the carbachol-control and experimental groups compared to AUC values of vehicle-control group during the early and late phases. Finally, two-way ANOVA followed by Bonferroni›s test was used to determine the effect of time (early or late phase of formalin-induced pain) and treatment on the percentage changes of AUCs of treatment groups compared to the AUC values of saline group. *P*-values less than 0.05 were considered to be statistically significant.

## Results

*Effect of intra-accumbal administration of OX1 receptor antagonist on antinociception induced by chemical stimulation of the LH*

**Figure 2:**
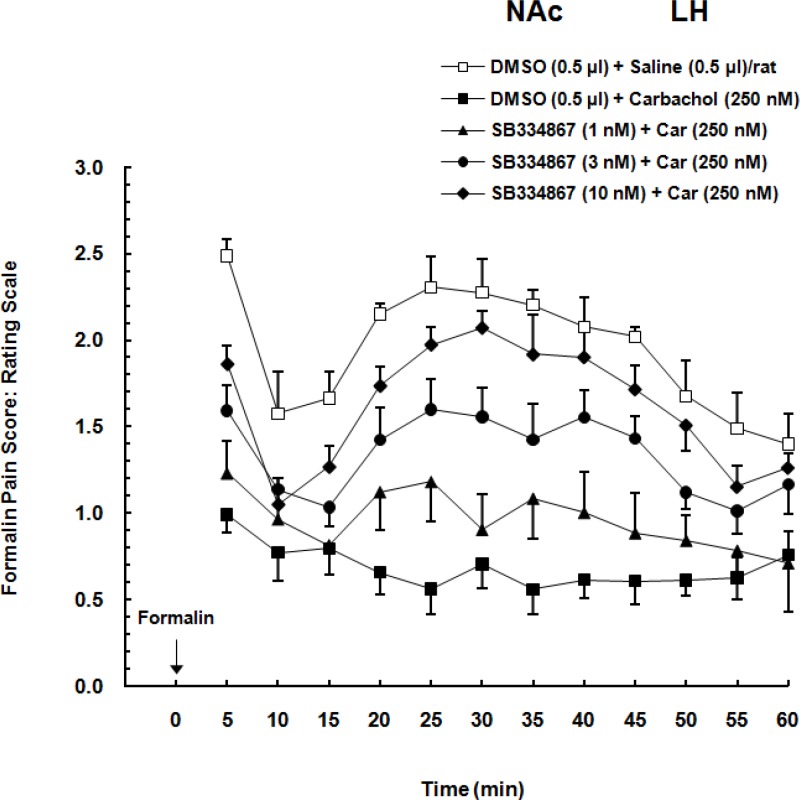
Effect of intra-accumbal (NAc) administration of different doses of SB334867 (1, 3 and 10 nM/0.5 µL DMSO), a selective OX1r antagonist, on antinociceptioninduced by chemical stimulation of the lateral hypothalamus (LH) in the formalin test. The average of pain scores (pain behaviors) in 60-min period after formalin injection is shown in this Figure. Carbachol-control group received 0.5

Two-way ANOVA followed by Bonferroni’s test [Treatment effect: F (4,308) = 19.19, *P<*0.0001; Time effect F (11,308) = 9.592, *P<*0.0001; Interaction F (44,308) =1.483, *P *= 0.0708] showed that administration of different doses of OX1r antagonist, dose-dependently decreased the LH stimulation induced analgesia in the form of an increase pain scores in formalin test. In comparison to the carbachol-control group, SB334867 could attenuate the analgesia induced by chemical stimulation of the LH especially in higher doses (3 and 10 nM) and this attenuation continued during the 60-min period of the formalin test. As it is shown in Figure 2. in vehicle-control group formalin administration cause biphasic pain and illustrated as an increase in pain scores. In comparison with vehicle-control group, carbachol injection decreased the pain scores. Intra-accumbal injection of different doses of OX1 antagonist before administration of carbachol into the LH, dose-dependently attenuated the LH stimulation-induced analgesia in the form of an increase in pain scores.

µL DMSO into the NAcbefore injection of carbachol (250 nM; intra-LH). In vehicle-control group animals received 0.5µL DMSO into the NAc and saline into the LH. Each point shows the mean ± S.E.M. for 5-8 rats.

**Figure 3:**
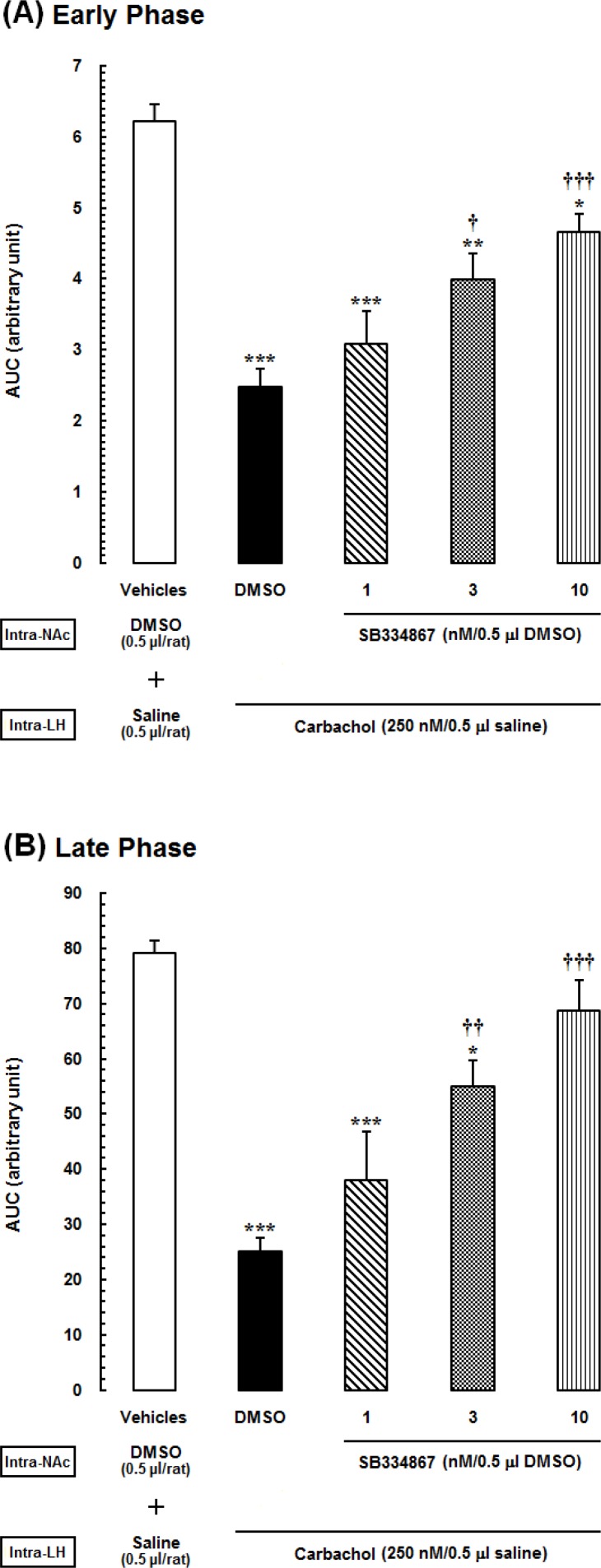
Area under the curves (AUCs) calculated separately for pain scores shown in the previous Figure. *(A)* Early (5 min), and *(B)* late (15–60 min) phases of formalin-induced pain, during 60-min period of the test. Animals received intra-accumbal injection of SB334867 (1, 3 and 10nM/0.5Μl DMSO) prior to administration of intra-LH carbachol (250 nM), then tested for formalin test.

*P<0.05, **P<0.01, ***P<0.001 different from the vehicle-control group

†*P*<0.05, ††*P*<0.01, †††*P*<0.001 different from thecarbachol-control group.99

**Figure 4:**
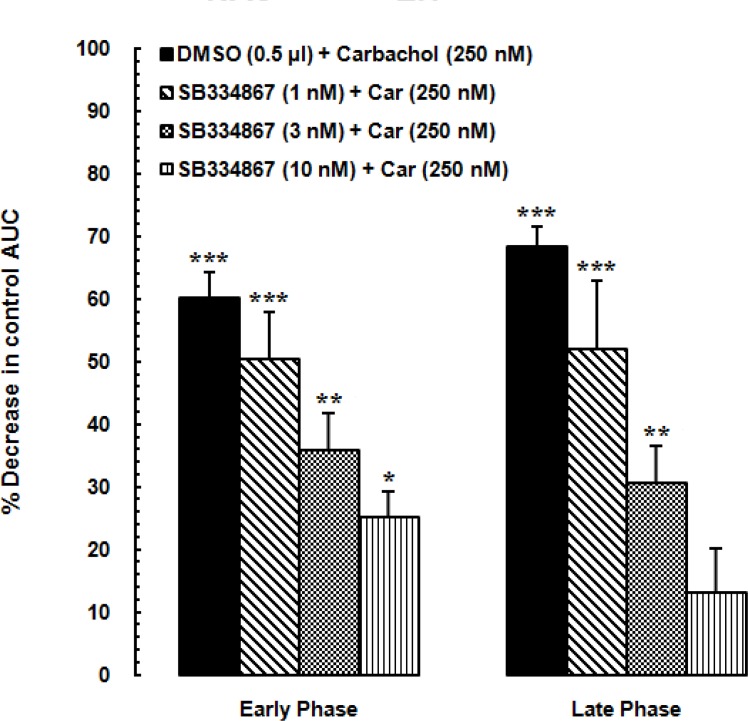
Diagram showing percentage decrease in the area under the curve (AUC) values calculated for treatment groups compared to the AUC value calculated for the vehicle-control group. SB334867 (1, 3 and 10 nM/0.5μL DMSO) or 0.5 μL DMSO injected into the nucleus accumbens (NAc) before administration of the carbachol into the LH. The AUC values calculated for treatment groups were normalized by that of the vehicle-control group.

*P<0.05, **P<0.01, ***P<0.001 different from the vehicle-control group.

**Figure 5:**
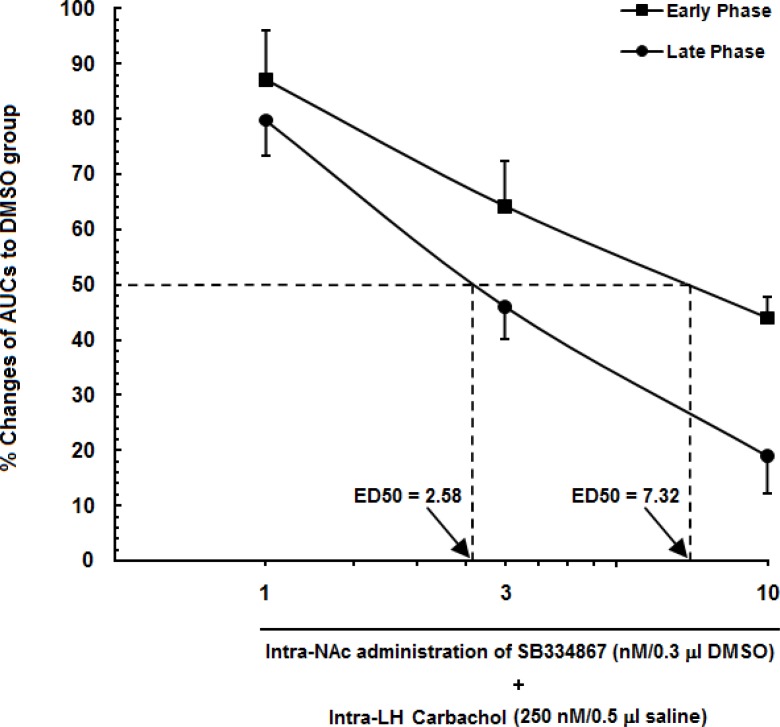
A log dose–response curve for carbachol in the lateral hypothalamus (LH) and SB334867 as an OX1r antagonist into the nucleus accumbens (NAc) in either the early phase of formalin-induced pain or the late phase. In this figure, we set the area under the curve (AUC) of the carbachol-control group (animals that received only 250nMcarbachol in the LH) to 100%, and represent the remaining AUC (animals that received different doses of SB334867 into the NAc) as a % changes in their effects to create an effective dose 50% (ED50) of SB334867 in the NAc in early (7.32) and late (2.58) phases.

*Effect of intra-accumbal administration of SB334867 on antinociception during the early and late phases of formalin-induced pain*

The acquired AUC values of each experimental group during the early and late phases were compared with those calculated for vehicle- and carbachol-control groups. In Figure 3. one-way ANOVA followed by Newman-Keuls test illustrated that carbachol analgesic effect, represented as a reduction in AUC values compared to the vehicle-control group (*P*<0.001). Furthermore, administration of different doses of SB334867, (1, 3 and 10 nM/0.5 μL 12% DMSO), dose dependently decrease the LH stimulation induced analgesia significantly compare to carbachol-control group in the early phase [F (4,32) =17.23, P<0.0001; Figure 3A.] and in the late phase [F (4,32) = 16.11, P<0.0001; Figure 3B.]. This effects shown as an increase in AUC values during both phases. However, in both early and late phases, this effect was seen in the groups which received the higher doses of SB334867 (3 and 10 nM) and the lowest dose (1nM) did not have any effect on the analgesia induced by LH stimulation.

*AUC calculated values of experimental groups after intra LH-administration of carbachol*

In Figure 4. AUC values of vehicle-control group is considered as zero. Using one-way ANOVA followed by Dunnett*ꞌ*s test percentage decrease of AUC values of carbachol-control and experimental groups were compared to the AUC values of vehicle-control group during the early phase [F(4,32) =18.33, *P*<0.0001; Figure 4. Right panel] and late phase [F (4,32) = 16.43, *P*<0.0001; Figure 4. Left panel]. SB334867 administration at the dose of 1, 3 and 10 nM made a reduction in percentage of decrease of AUC values compared to the carbachol-control group. The least percentage of decrease was observed in the group which received 10 nMSB334867 and the effect of SB334867 administration at the dose of 10 nM on reduction of antinociceptive effect of carbachol was more in the late phase than the early phase. 

In Figure 5. a log dose-response curve for carbachol and SB334867 into the LH and NAc has been shown. The AUC values of carbachol-control group set as 100% and the AUC values of experimental groups were compared to those of the carbachol-control group. Percentage of changes of AUC values of rats which had received SB334867 at the doses of 3 and 10 nM were less in the late phase than those of the early phase. Furthermore, this figure shows that the 50% effective dose (ED50) value of SB334867on LH-induced antinociception in the late phase (2.58 nM) is less that in the early phase (7.32 nM). Altogether, it seems that contribution of OX1 receptors in modulation of LH-induced analgesia in the late phase is more than that in the early phase.

## Discussion

Our purpose in this study was to evaluate the involvement of OX1 receptors within the NAc in the antinociceptive responses induced by LH stimulation in formalin test as an inflammatory model of pain in rats. Our results showed that unilateral intra-accumbal microinjection of different doses of SB334867, OX1r antagonist, attenuated the antinociception induced by chemical stimulation of LH by carbachol during both phases of the formalin test. Moreover, as shown in our results, the effect of blockade of OX1 receptors on reduction of antinociception was more in the late phase rather than the early phase.

Former studies have shown that the chemical and electrical stimulation or inactivation of the LH can produce antinociception in acute and tonicmodels of pain in rats (11, 16, 21-23). Role of orexinergic system on pain modulation has been investigated in several previous studies. For example, it has been shown in a study by Jeong and Holden, 2009 that administration of orexin A systemically produced naloxone insensitive antinociceptive effects on pain and they found that this effect is partly mediated by OX1 receptors in the central nervous system (24). Furthermore, intrathecal administration of orexin A indicated to be inhibiting pain perception in behavioral tests (6, 25). Moreover, orexin A has been tested for the hot-plate, tail-flick, and formalin tests in mice and shown that it has analgesia effects (26). Working with orexin knockout mice showed that they presented higher degree of hyperalgesia induced by peripheral inflammation than the wild type mice (27). The orexinergic neurons and their fibers as well as OX1 and OX2 receptors have been shown to be distributed through the pain circuitry including periaqueductal gray matter (PAG) region which is considered to be important for pain modulation (28, 29). Azhdari-Zarmehri *et al*. (2014) demonstrated that the antinociceptive effects induced by injection of orexin A into the rostroventral medulla (RVM), blocked by administration of selective orexin-1 receptor antagonist, SB334867, in the formalin test (30). 

We previously showed in our laboratory that antinociception induced by chemical stimulation of the LH attenuated by the blocked of OX1 and OX2 receptors within the VTA and NAc in the tail-flick test (4, 5). Furthermore there is evidence of the involvement of the orexinergic system within the PAG and RVM in pain modulation. They showed that injection of orexin A into these areas attenuated the formalin nociceptive behavioral (9, 30). All the studies mentioned are in agreement with the results of our study. Together, it seems that the LH modulation in pain works through the orexinergic system within the VTA and NAc in the brain steam, and also trough the RVM and PAG. 

Therefore our data are in line with the findings of previous works and it is showed that intra-accumbal OX1 receptors are involve in formalin-induced pain during the early and late phases. However, the effects of blockade of these receptors during the late phase were more than the early phase. This difference can be attributed to the different mechanisms underlying the development of both phases. In this respect, Wheeler-Aceto *et al*. (1990) and TjØlsen *et al*. (1992) have suggested that the early phase is caused by toxic and destructive effects of formalin on tissue around the injection site and repetitive firing of C-fiber nociceptors; and the second one is due to the inflammatory response to the tissue damage as well as long-term changes in the response of dorsal horn neurons to C-fiber input that increase gradually (wind up) (31, 32). Previous results showed that intra-LH administration of carbacholreduces both phases of formalin-induced pain but antinociceptive effect of carbachol is more pronounced during the late phase (21). Similarly, it has been shown recently that orexin antagonists within the VTA reducedthe LHinducedantinociception during both phases and this Anti-analgesic effect was moreconsiderable during the late phase (33).Accordingly, in this study, the effect of orexin receptor antagonist administration into the NAcon reduction of antinociceptive effect of carbacholwas more during the late phase than the early phase. Formalin predominantly increase the activity of C-fibers not Aδ fibers (31). So, it seems that orexinergic system is more involved in regulation of mechanisms underlying the development the central sensitization. However, studying the molecular mechanisms or molecular changes involved in central sensitization and the effect of orexinergic system on central sensitization were out of the scope of this study. Also, molecular studies are strongly recommended. It has been supposed that the NAc mediates the antinociception and facilitate nociception through the RVM as well as through the spinal cord level (34). However, more pharmacological and electrophysiological experiments are needed to understand the role of the orexinergic system in the pain modulation at the level of supraspinal and spinal cord. 
